# Machining Accurate Deep Curved Forms on Tungsten Carbide–Cobalt (WC-Co) Eliminating Tool Wear in the Electrical Discharge Turning Operation

**DOI:** 10.3390/mi16101167

**Published:** 2025-10-15

**Authors:** Mohammadjafar Hadad, Mehdi Soleymani, Amir Alinaghizadeh

**Affiliations:** 1 Department of Mechanical Engineering, College of Engineering and Technology, University of Doha for Science and Technology, Doha P.O. Box 24449, Qatar; 2 School of Mechanical Engineering, College of Engineering, University of Tehran, Tehran P.O. Box 14155-6619, Iran; soleimani.mehdi@ut.ac.ir; 3 Institute for Advanced Manufacturing (KSF), Furtwangen University, 78532 Tuttlingen, Germany; aam@hs-furtwangen.de; 4 Energy and Sustainable Development Research Center, Semnan Branch, Islamic Azad University, Semnan P.O. Box 35145-179, Iran

**Keywords:** electrical discharge turning, tungsten carbide–cobalt, tool wear, surface roughness, form machining

## Abstract

Machining hard metals presents various challenges, especially with materials like WC-Co, known for their exceptional hardness and wear resistance, making them ideal for cutting tools. Among machining methods, Electrical Discharge Machining (EDM) stands out for its ability to machine hard materials with no mechanical damage, which is critical for machining fragile components. For form shape machining symmetrical parts like WC-Co bars, electrical discharge turning (EDT) could be applied. Despite its potential, limited research exists on deep form turning of hard metals like WC-Co using EDT. This study addresses that gap by comparing the final geometrical outcomes of two EDT setups: vertical and horizontal tool electrode configurations. Additionally, the impact of workpiece rotational speed on surface quality was examined. Results showed that the vertical tool electrode setup produced more accurate geometries and smoother surfaces. Furthermore, increasing the workpiece’s rotational speed improved flushing efficiency, resulting in reduced surface roughness and a cleaner machined surface.

## 1. Introduction

Machining hard-to-cut materials is a field of significant interest because, despite the various challenges involved, their excellent mechanical properties make them valuable in many industries [1]. Tungsten carbide–cobalt (WC-Co) is a well-known hard material, recognized for its high corrosion and erosion resistance [2]. The high wear resistance of tungsten-based composites has expanded their applications in cutting tools, metal forming tools, the drilling industry, and even jewelry-making [3,4,5]. One of the earliest nonconventional processes is Electrical Discharge Machining, which has a wide range of applications in the machining industry [6]. Electrical Discharge Machining is particularly suitable for machining hard metals and superalloys because it is not affected by the hardness and machining difficulty of these materials [7]. Although the die-sinking process is the most common form of EDM, the need to machine round symmetrical parts such as metallic bars has led to the emergence of new EDM-based processes, such as EDT. In fact, EDT is a hybrid machining process that combines conventional turning and EDM to machine round symmetrical parts.

Despite the high potential of the EDT process, it has not been thoroughly investigated. Only a few studies have been conducted on this topic. In particular, research focusing on form machining of hard metals using EDT is rare. In 2001, Guu studied the machining of AISI D2 tool steel using a rotational EDM setup [8]. In 2008, Matoorian optimized the EDT parameters to arrive at the best performance when machining High Speed Steel (HSS) bars by EDT [9]. In 2016 and 2018, Gohil applied EDT to machine the SS-304 stainless steel bars [10,11]. In 2016, 2015, and 2018, Gohil studied EDT on Ti-6Al-4V [12,13,14]. Also, in 2017, Puri investigated the machining of Ti-6Al-4V bars by EDT to study the Material Removal Rate (MRR) [15]. In 2019, Azhiri investigated the effect of EDT input parameters on the MRR and residual stress during the machining of AISI D2 bars by EDT [16]. In addition, in 2020, Azhiri investigated surfaces of AISI D2 machined by EDT under an applied magnetic field [17]. In another study on the EDT process in 2020, Chakiroglu investigated machining AISI L2 bars by EDT. The MRR, surface roughness, and Tool Wear Ratio (TWR) were investigated [18]. Also in 2020, researchers studied the machining of AISI D2 bars by EDT, applying a magnetic field to produce straight grooves [19]. In 2021, the circularity deviation, TWR, and surface roughness were studied in machining of mild steel bars by EDT [20]. EDT was investigated in 2021, and MRR, Over Cut, surface roughness, Recast Layer Thickness (RLT), and hardness were studied when machining the die steel AISI D2 [21]. The surface integrity and fatigue life of Inconel 718 bars were studied when machining them by EDT in 2021 [22]. Although no prior literature was found in relation to the electrical discharge turning of WC-Co applying rigid tool electrodes, in an investigation, wire-cut Electrical Discharge Machining was used in the turning operation of WC bars. This study analyzed the Material Removal Rate and surface roughness, considering the pulse on-time, pulse off-time, wire feed, and wire tension as the input parameters. Optimization using Taguchi and ANOVA methods revealed that pulse on-time and pulse off-time were the most significant parameters to arrive at the highest Material Removal Rate and the lowest surface roughness [23]. However, this study did not study the effect of the rotational speed on the machining results.

A review of the literature reveals three significant gaps in studies related to the EDT process. The first gap is that most investigations have focused on mild steels, titanium alloys, tool steels, and HSS, while no studies have examined super-hard materials like WC-Co. This is notable because of the increasing industrial demand for high-hardness materials like WC-Co. Secondly, existing studies on EDT have primarily involved shallow machining, involving low feed rates and minimal depths of cut (mostly limited to a few tenths of a millimeter), likely intended to reduce machining time and avoid technical complications. However, many practical applications involving symmetrical machined bars require deeper machining. The third gap is the lack of studies exploring the machining of curved forms using the EDT process. To address these gaps, this study investigates the deep machining of curved geometries on WC-Co composites using the EDT process.

Additionally, since undesirable Tool Wear Ratio (TWR) is an inherent issue in EDT that affects geometrical accuracy, this study compares two different EDT setups (vertical and horizontal) for machining curved forms. We propose a setup that significantly mitigates the impact of TWR on the final geometrical accuracy. Moreover, the resulting geometries and surface integrity obtained from the two EDT setups are compared with each other and with those achieved using conventional EDM.

## 2. Material and Methods

**Figure 1:**
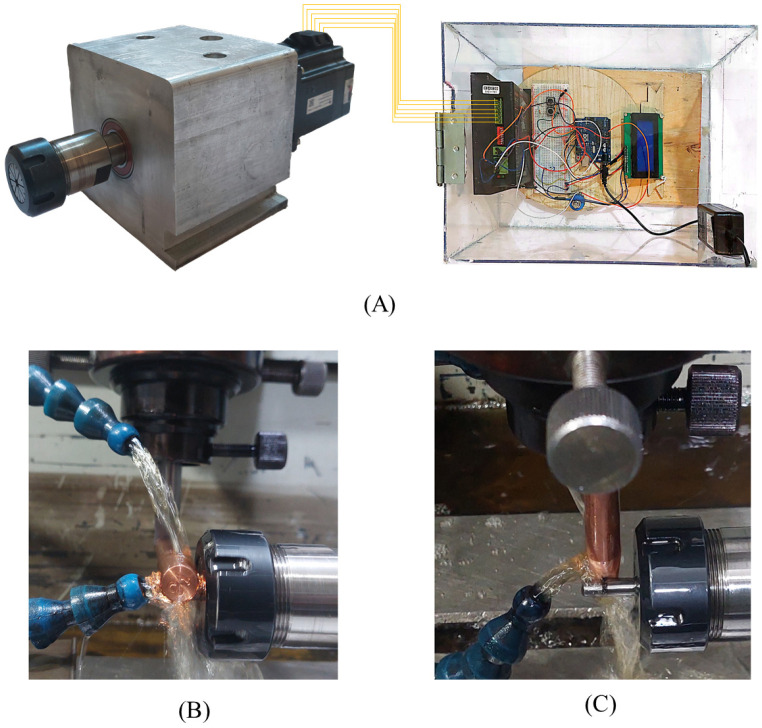
The rotating mechanism (**A**), the horizontal EDT setup (**B**), and the vertical EDT setup (**C**).

The workpiece material used in this study was WC-Co bars with a diameter of 6 mm, and their composition is listed in Table 1. The WC-Co workpieces were selected from h6 tolerance bars, which are used to manufacture the WC-Co end mills. A cylindrical copper bar with a diameter of 12 mm was used as the tool electrode. The copper bars were cut into 200 mm lengths. Three pieces were used as the tool electrodes for the horizontal setup, and three other pieces were used for the vertical EDT setup. Also, a smooth conical shape was machined on one end of the three tool electrodes used for the vertical EDT setup to make them ready for gradual engagement in EDT machining, thereby promoting more consistent results. The rotational speed of the workpiece was precisely controlled by a stepper motor, which was driven by a microcontroller and a motor driver (Figure 1A).

One of the critical requirements in such a machining process is precise controlling of the geometrical sets. For example, in both vertical and horizontal setups, the tool electrode’s central axis should be exactly perpendicular to the workpiece’s central axis. Improper alignment can lead to instability of the machining process or geometrical errors on the final machined forms.

Also, as depicted in Figure 1, the dielectric was provided using hoses with oil fluid at the rate of 4 l/min and all the EDT experiments were conducted applying an iso-pulse EDM machine.

**Figure 2:**
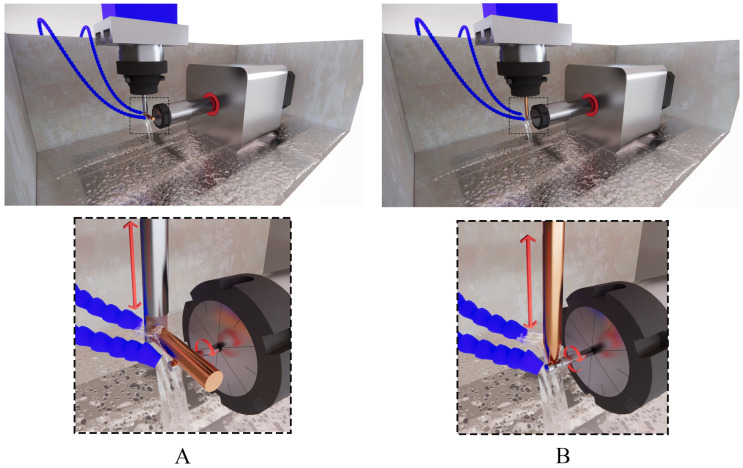
The schematic of the setups, (**A**): the horizontal EDT and (**B**): the vertical EDT.

In order to machine a circular curve on the WC-Co bars, two setups were considered. In the first setup, the axis of the tool electrode was parallel to the machine table (Figure 1B); this setup is referred to as the horizontal setup. In the second setup, the axis of the tool electrode was perpendicular to the machine table (Figure 1C); this setup is referred to as the vertical setup. Also, Figure 2 illustrates the main components and their relative position in the two EDT setups schematically.

## 3. Results and Discussion

This section presents the results of the current research. Section 3.1 presents the results of machining the curve forms on WC-Co parts by horizontal and vertical EDT. Since the primary distinction between traditional EDM machining and the EDT process is caused by the existing rotational movement in the workpiece, we precisely compared the results between horizontal and vertical EDT by varying the workpiece’s rotational speed from 5 rpm to 20 rpm. All other machining parameters such as pulse on-time, pulse off-time, discharge current, and other parameters remained constant, and the set values are shown in Table 2. Additionally, a separate set of experiments was conducted (in Section 3.2) to compare the machining results in three different regimes including roughing, semi-finishing, and finishing. The variable parameters included depth of cut (defined as the radial portion of the tool electrode engaging in the machining process) which varied from 0.1 mm to 0.5 mm, rotational speed (between 0 and 45 rpm), discharge current (between 1 and 4 A), and pulse on-time (between 150 µs and 300 µs). The rest of the machining parameters were kept constant and are listed in Table 3.

### 3.1. Horizontal and Vertical EDT

**Figure 3:**
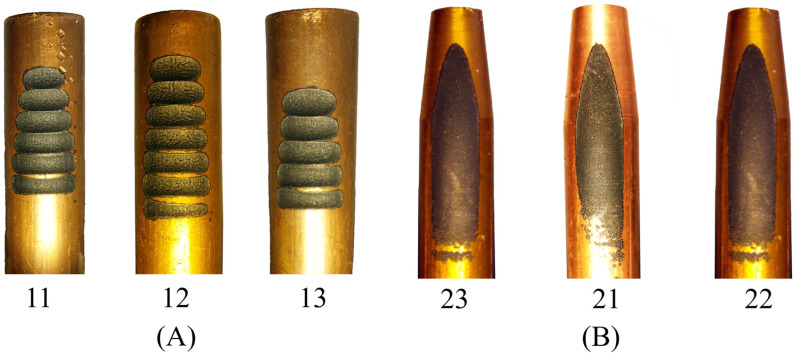
The tool electrodes of the horizontal EDT setup and their number based on Table 2 (**A**), and the tool electrodes of the vertical EDT setup (**B**).

In the horizontal setup, the erosion of the tool electrode can lead to geometrical deterioration of the machined workpiece. To reduce its harmful effect on the final geometry, we attempted to shift the eroded part of the tool electrode to a fresh area. At the beginning of machining with the horizontal setup, electrical discharges were more concentrated on a smaller area (as shown in Figure 3A), resulting in a shorter eroded region of the tool electrode compared to the area eroded later. Since in machining by the horizontal setup there is no exact sign to detect when the desired circular form has been machined (due to simultaneous erosion of both the tool electrode and the workpiece), we changed the position of the tool electrode every 10 min to replace the tool’s eroded area with a new fresh region. In contrast, in machining by the vertical setup, we observed no electrical discharges as soon as the circular part was machined completely based on the preset depth of cut. So, the form and size of the machined area could be controlled.

In contrast, in the proposed vertical EDT setup, the tool electrode included a conical section at its tip, allowing it to gradually engage with the machining gap. Over time, the engaged portion of the tool electrode expanded until it reached the maximum depth of machining, after which the tool electrode passed through the workpiece without further discharges, indicating that machining was complete. The advantage of the vertical EDT setup is that tool electrode erosion becomes less of a concern, as the worn section (with undesirable geometry) is automatically replaced by a fresh region. This eliminates the negative impact of electrode wear on the final machined geometry. Therefore, the vertical EDT setup is an excellent choice for machining various form shapes, regardless of their geometry or depth.

However, when using the horizontal EDT setup, simultaneous and uncontrolled erosion of both the workpiece and the tool electrode makes it extremely difficult to determine the correct timing or position (i.e., the relative alignment between the workpiece and electrode) to stop the machining process.

**Figure 4:**
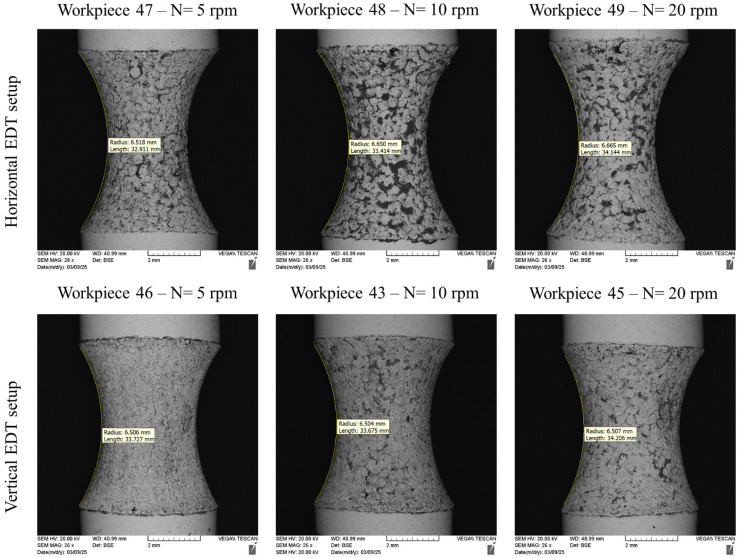
The arc forms machined by horizontal EDT, the workpiece number based on Table 2, and the corresponding arc radiuses.

The analysis of the arc shapes machined on the WC-Co workpieces was conducted using Digimizer software (Version 6) [24]. We fitted the nearest arc to the machined form’s shape to evaluate the corresponding radii, as shown in Figure 4. For this purpose, we first calibrated the Digimizer measurement tool by the corresponding scale bar of each image; then, the resulting radius of EDT machining was obtained by fitting the circular curve on the machined area.

The results revealed that the arcs machined by the horizontal EDT setup had larger radii than those machined by the vertical EDT setup. Additionally, the radii of the arcs machined by the horizontal EDT setup varied as the rotational speed changed.

When machining with the horizontal EDT setup, a negative effect occurred, which undesirably influenced the form shapes. This effect was high tool electrode erosion, which led to a deterioration of the circular shape of the tool electrode, increasing the radius of the eroded area. As the radius of the tool electrode increased, the machined arcs on the workpieces also had larger radii. Furthermore, arcs machined at higher workpiece rotational speeds (in the horizontal EDT setup) exhibited larger radii. Increasing the rotational speed improved the flushing, making the machining gap cleaner and increasing the gap discharge voltage. This allowed for more powerful discharges to occur, leading to greater tool electrode erosion.

On the other hand, machining the arc forms with the vertical EDT setup resulted in consistency in the radii of the machined forms, as illustrated in Figure 4. Although increasing the rotational speed of the workpieces improved the flushing in the machining gap, leading to higher tool electrode erosion, the high tool electrode erosion did not affect the machined geometry. This is because the eroded area was continuously replaced by a fresh region of the tool electrode in the vertical EDT setup. Therefore, changes in the rotational speed of the workpiece and tool electrode erosion did not influence the final arc form geometry.

Overall, machining WC-Co bars using the horizontal EDT setup resulted in noticeable inconsistency between the intended radius and the actual machined radius, as illustrated in Figure 4. The final forms produced using the horizontal EDT setup exhibited smaller and less uniform radii compared to those produced with the vertical setup. This discrepancy arises primarily due to the lack of control over the machining geometry in the horizontal configuration, where simultaneous erosion of both the tool electrode and the workpiece leads to unpredictable material removal and dimensional inaccuracies.

**Figure 5:**
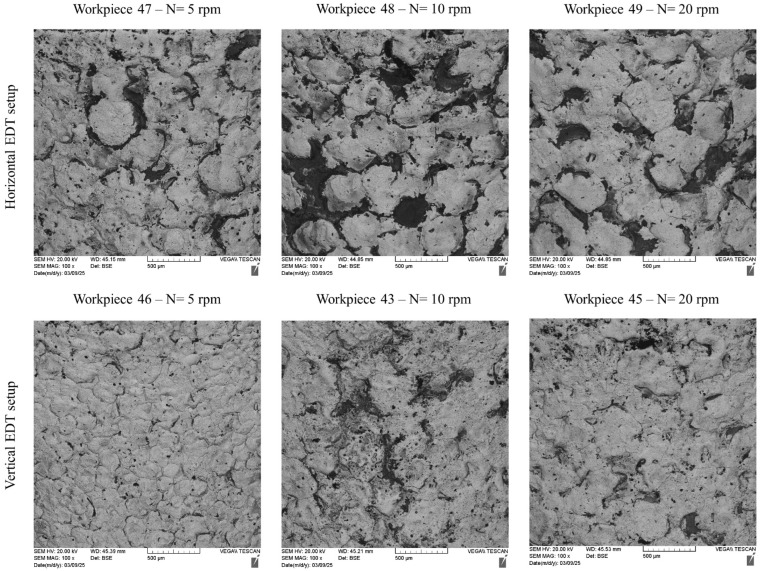
The machined surfaces with various rotational speeds after vertical and horizontal EDT.

**Figure 6:**
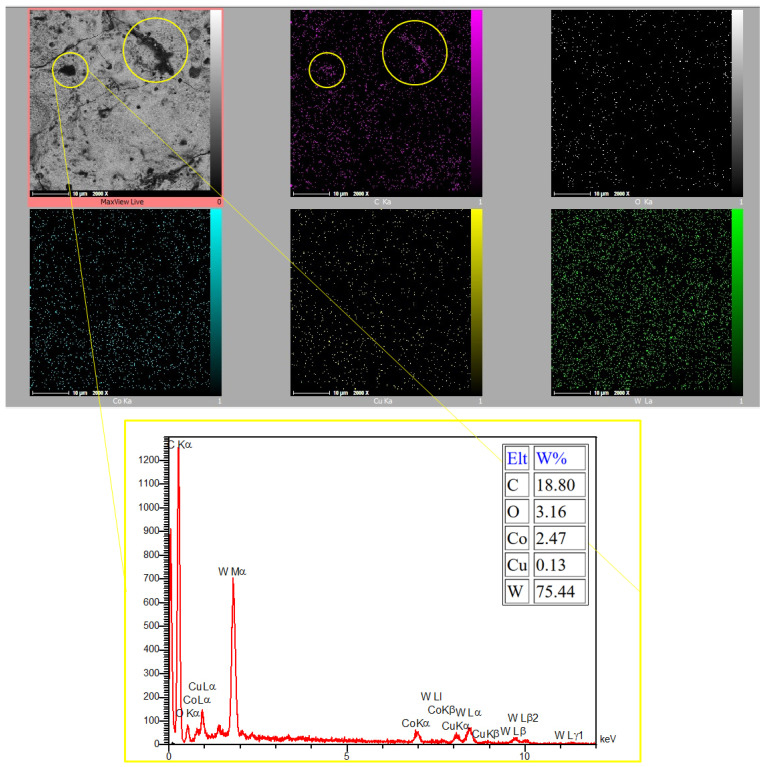
The EDS and Elemental Mapping results on the high-carbon area of the machined surface.

Figure 5 shows the magnified machined surfaces after vertical and horizontal EDT. As depicted, the three surfaces machined by vertical EDT generally exhibit cleaner surfaces with less remaining debris on them. In contrast, the three surfaces machined by horizontal EDT show areas filled with carbon-based phases. The carbon element was released either from the oil-based dielectric or from the WC-Co workpiece. Figure 6 presents the distribution and percentage of different elements on the machined surface using Elemental Mapping and EDS tests, highlighting a high concentration of carbon in the circled areas.

In the vertical EDT setup, the gap between the tool electrode and the workpiece is aligned with the direction of gravity, which acts on the molten material suspended in the dielectric. As a result, gravity aids in the evacuation of the residuals, leading to improved flushing and cleaner machined surfaces.

### 3.2. Vertical EDT in Roughing, Semi-Finishing, and Finishing

A comparison was conducted between the machining results of vertical EDT under roughing, semi-finishing, and finishing conditions. Each of the workpieces—71, 72, and 73—was machined using one of the three regimes, and the results were then compared. For rough machining, the machining depth of cut was set to 0.5 mm, the discharge current to 4 A, and the pulse on-time to 300 µs. For semi-finishing, the machining depths of cut were set to 0.3 mm and 0.2 mm, with discharge currents of 4 A and 2 A and pulse on-times of 300 µs and 150 µs, respectively. For finishing, the machining depths of cut were 0.25 mm, 0.15 mm, and 0.1 mm, with corresponding discharge currents of 4 A, 2 A, and 1 A, and pulse on-times of 300 µs, 150 µs, and 50 µs.

In order to implement the roughing process, the depth of cut was set to 0.5 mm, meaning that the tool electrode was set to 0.5 mm to be engaged with a workpiece in its radial direction. However, in semi-finishing process, first, the workpiece was machined by a depth of cut of 0.3 mm. After completing the machining and creating the round shape with 0.3 mm indentation, the depth of cut was added by 0.2 mm to complete the semi-finishing process on the same workpiece. Also, in the finishing process, we applied a similar process, but the depths of cut were set to 0.2 mm, 0.15 mm, and 0.1 mm simultaneously. As mentioned, at each stage, the discharge current and pulse on-times were changed corresponding to the values in Table 3.

Two different rotational speeds—0 and 45 rpm—were considered to examine the effect of rotation on machining performance in the roughing, semi-finishing, and finishing modes. Since machining with zero rotational speed in EDT is effectively the same as traditional EDM, this experiment also served as a comparison between EDT and conventional EDM. Table 3 presents the experimental details for the machining process across the three machining regimes.

**Figure 7:**
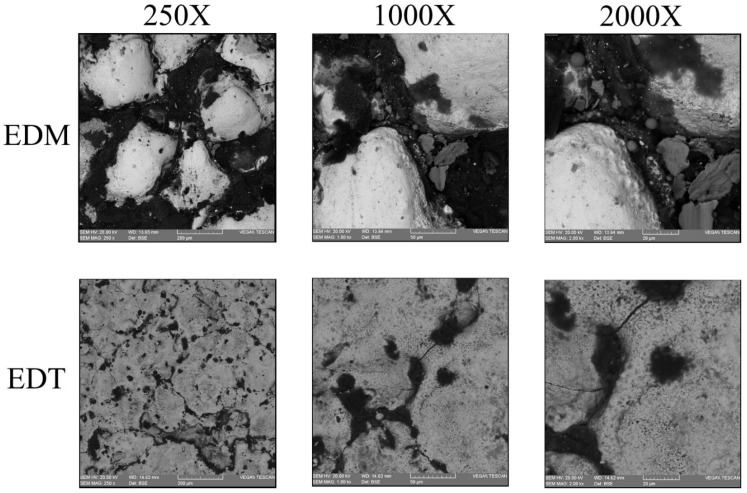
The rough machined surfaces after vertical EDT and EDM.

**Figure 8:**
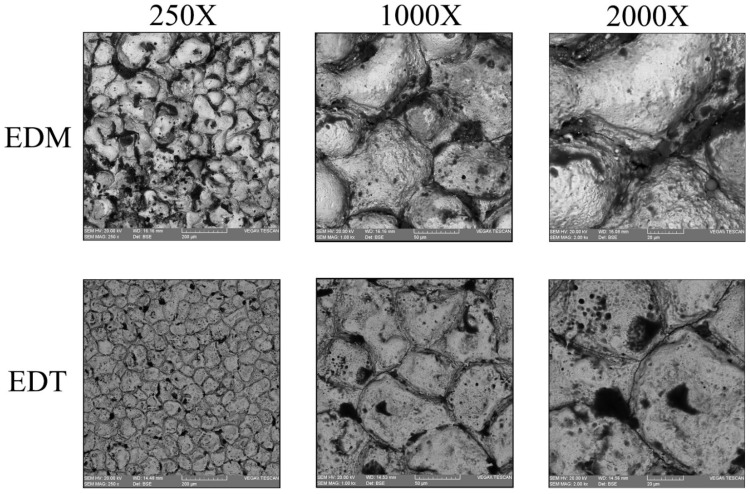
The semi-finished surfaces machined by vertical EDT and EDM.

**Figure 9:**
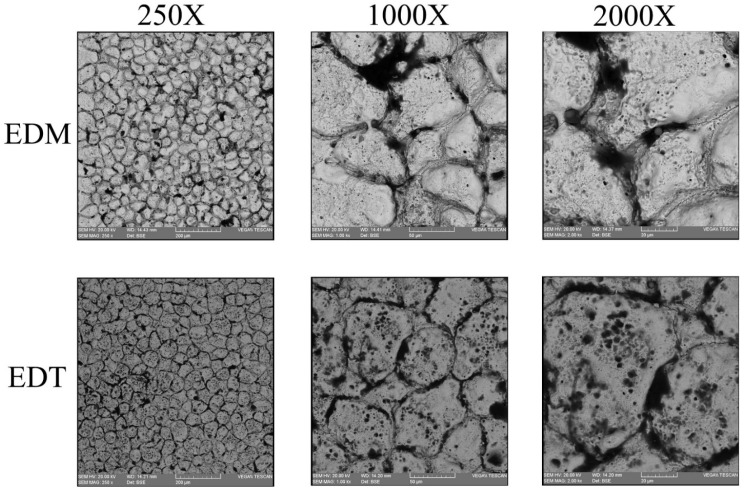
The finished surfaces machined by vertical EDT and EDM.

Figure 7, Figure 8 and Figure 9 illustrate the machined surfaces produced by the vertical EDT and EDM processes under roughing, semi-finishing, and finishing regimes, respectively. Reducing the workpiece’s rotational speed from 45 rpm to 0 resulted in an accumulation of residuals on some spots on the machined surfaces due to reduced flushing performance. This effect is more noticeable in the roughing regime (Figure 7). However, the surface textures in the finishing regime remain nearly the same, as less molten material is generated during finishing, and high-performance flushing (due to higher rotational speed) is not required for effective debris removal.

**Figure 10:**
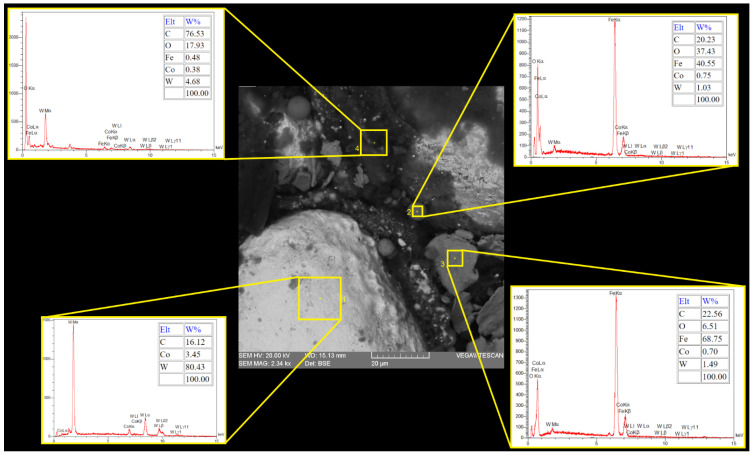
The element percentage of the different areas and particles on the EDMed surface.

Further investigation revealed that the flushing performance of the EDM process (with no rotation) was even lower than initially expected. Some particles were found on the machined surfaces that did not originate from either the workpiece or the tool electrode; this is particularly evident in Figure 7 at 2000× magnification. EDX tests confirmed that these particles contained a high amount of Fe element (Figure 10). Since Fe is not present in either the WC-Co workpiece or the copper tool electrode, the source of these particles was identified as contamination from the dielectric fluid, which had previously been used to machine Fe-based materials. Due to the poor flushing performance, these foreign particles entered the machining gap from the dielectric and remained on the machined surface. Although the pre-contamination of the applied dielectric did not affect the machined surfaces by the EDT process due to its good flushing, the dielectric quality has a specific effect when machining by traditional EDM.

**Figure 11:**
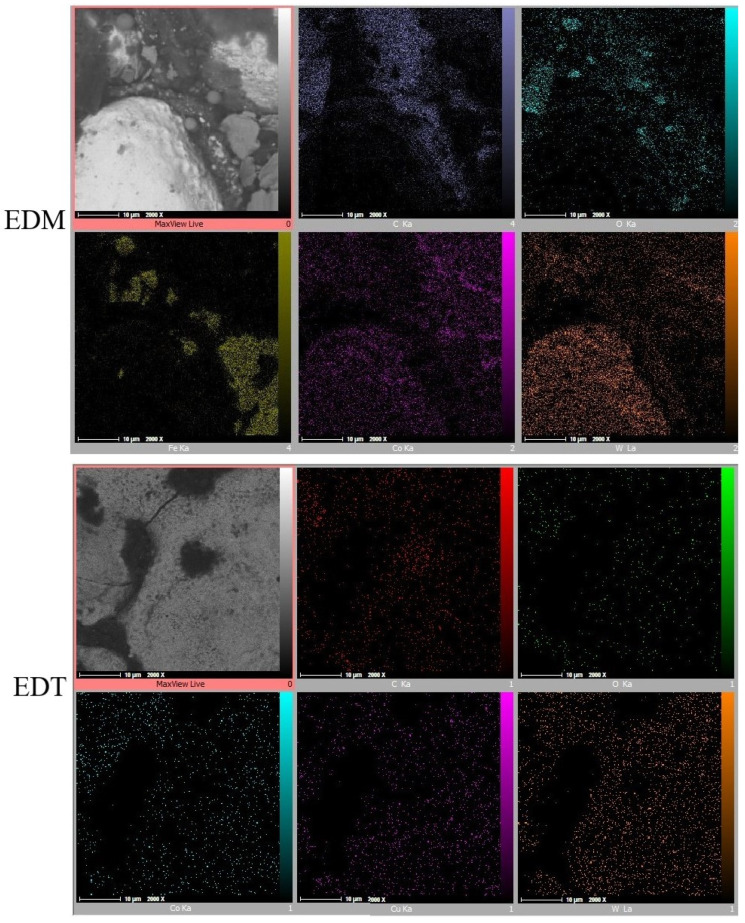
The elements’ distribution on the machined surfaces by vertical EDT and EDM in the roughing mode.

**Figure 12:**
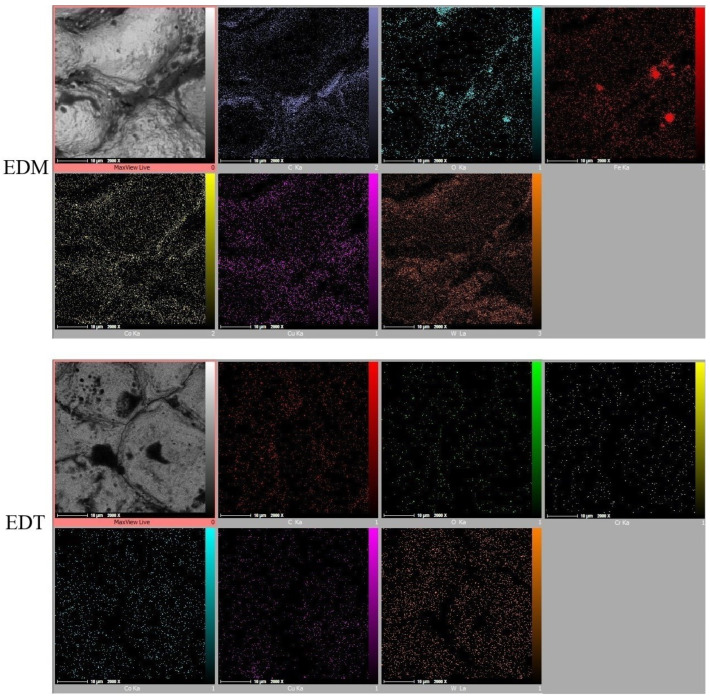
The elements’ distribution on the machined surfaces by vertical EDT and EDM in the semi-finishing mode.

**Figure 13:**
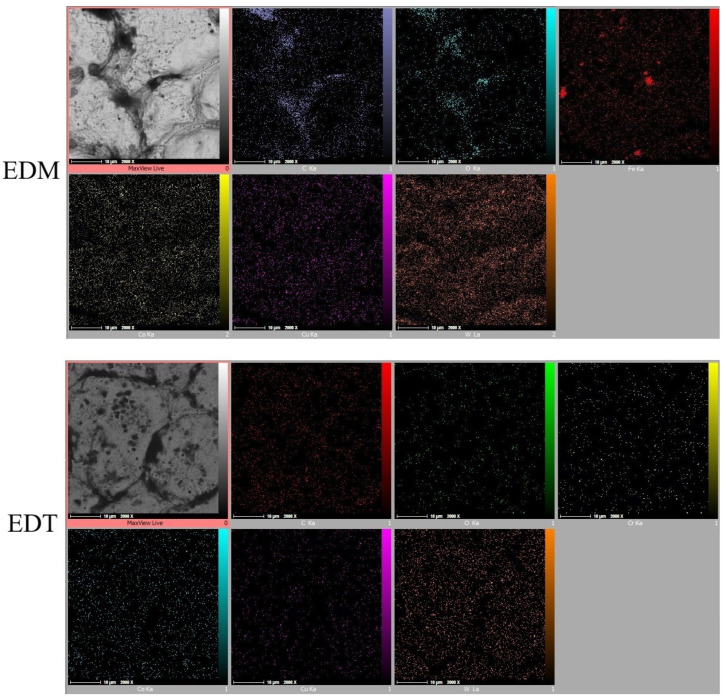
The elements’ distribution on the machined surfaces by vertical EDT and EDM in the finishing mode.

Elemental Mapping tests (Figure 11, Figure 12 and Figure 13) revealed that iron (Fe) is not the only element found in greater quantities on EDM-machined surfaces compared to those machined by EDT. While the elements detected on the surfaces machined by EDT were distributed relatively uniformly, several elements on the EDM-machined surfaces were found to be concentrated in specific regions. As previously mentioned, one of these elements is Fe, which originated from the dielectric fluid contaminated by previous machining operations on Fe-based materials.

In addition, oxygen and carbon were inconsistently distributed across the EDM-machined surfaces. Oxygen, which can enter the machining gap from the surrounding air, was observed either in Fe-based or carbon-based phases, indicating oxidation on the machined surfaces. The concentration of Fe, carbon, and oxygen was more prominent in the roughing regime due to the higher energy electrical discharges occurring during roughing.

Copper (Cu) was also detected on the machined surfaces, resulting from the migration of copper from the tool electrode into the machining gap. In contrast, the Elemental Mapping tests showed no significant accumulation of elements on the surfaces machined by EDT, indicating the high flushing performance achieved due to the rotational movement of the workpiece.

**Figure 14:**
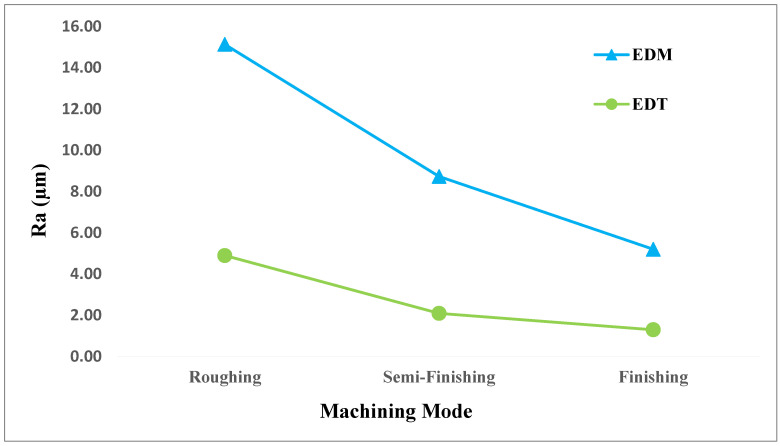
The comparison between the roughness of the machined surfaces by vertical EDT and EDM.

The surface roughness of the machined parts was also compared after machining with the EDT and EDM processes. Following the machining of the workpieces in roughing, semi-finishing, and finishing regimes, the resulting surface roughness was measured using a HOMMELWERKE Turbo Roughness Tester (V3.34). As shown in Figure 14, the surfaces machined by EDM were generally rougher than those machined by EDT. In the roughing regime, the difference in roughness values was significant, with the surface machined by EDM being 10.2 µm rougher than that produced by EDT (15.1 µm vs. 4.9 µm). In the semi-finishing regime, the difference was observed too: the roughness of the EDM-machined surface was 8.7 µm, while the EDT-machined surface had a roughness of 2.1 µm. Although both the processes resulted in low roughness values in the finishing regime, EDT still outperformed EDM, resulting in a surface roughness of 1.3 µm compared to 5.2 µm for EDM, showing a 3.9 µm improvement. Overall, the average surface roughness values resulted from the EDT process were about 67%, 75%, and 75% lower than the surface roughness resulting from the EDM process, showing a remarkable improvement in surface roughness when applying the EDT process.

The improved surface quality in EDT is attributed to its higher flushing performance enabled by the rotational movement of the workpiece. This enhanced flushing helps to remove molten material more effectively than in traditional EDM, preventing it from solidifying on the surface and thereby achieving better surface roughness.

**Figure 15:**
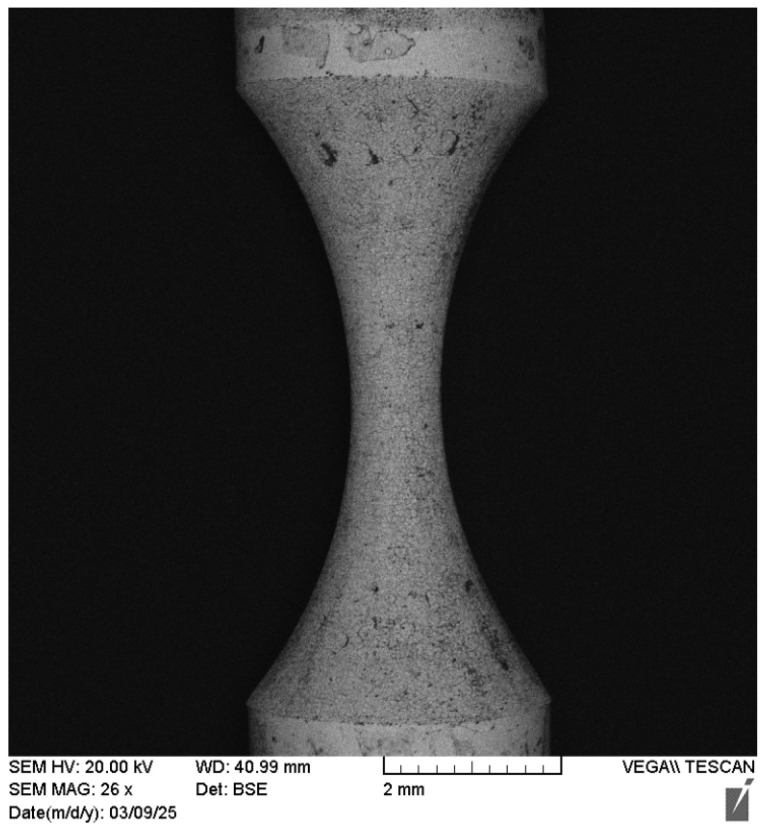
The curved form shape machined by vertical EDT in thinner workpiece and with higher aspect ratio.

The EDT process offers a reliable high-quality turning operation for conductive hard metals such as WC-Co. It can effectively machine various forms on hard, thin (low-radius) symmetrical parts, something that is nearly impossible using traditional turning methods. However, challenges such as limited control over the final machined geometry (due to significant tool electrode erosion) and relatively poor surface roughness can be addressed using the proposed technical solution known as vertical EDT. Vertical EDT enables the machining of complex, deep geometries regardless of the type or extent of tool electrode erosion. This is because the setup naturally compensates for the worn regions of the electrode by introducing fresh regions, eliminating the need for complex monitoring systems or advanced mathematical corrections. Figure 15 shows an example of a thin, brittle WC-Co bar on which vertical EDT successfully machined a high-aspect-ratio circular form.

## 4. Conclusions

To address the challenges of machining arc forms on WC-Co composites, the EDT process was employed, as it is capable of machining arc-shaped geometries on hard and fragile WC-Co bars. Two different EDT setups were evaluated including the vertical setup and the horizontal setup. The geometrical accuracy of the arc forms produced by EDT, along with the resulting surface integrity, was compared between these two setups. Additionally, the influence of rotational speed was investigated in relation to surface roughness and surface texture during vertical EDT machining under roughing, semi-finishing, and finishing regimes. This study proved the ability of EDT process in machining the accurate circular forms on cylindrical WC-Co bars while mitigating the adverse effects of tool electrode wear. However, more investigation is recommended to examine such a process in machining more complex forms or machining very thin and brittle bars. Also, artificial intelligence could be a beneficial tool to compensate the tool electrode wear when we are not able to apply vertical EDT. This method could be implemented using vision-based or signal-based intelligent algorithms. The key findings of this study are summarized as follows:

- Applying the vertical EDT resulted in significantly higher geometrical accuracy compared to the horizontal EDT when machining arc forms on WC-Co bars. In the horizontal EDT setup, tool electrode erosion adversely affected the final geometry on the final machined geometry. However, in the vertical EDT setup, the wear of the tool electrode had almost no negative effect on the final geometry, as the worn regions were continuously replaced by fresh regions during the machining process.
- The surfaces machined by the vertical EDT were cleaner and exhibited fewer residuals compared to those machined by the horizontal EDT. In the vertical setup, gravity is aligned with the machining gap, which aids in evacuating the extracted molten material from the machining area.
- The rotational speed applied in the EDT process improved the flushing performance. This improvement was more noticeable during roughing compared to semi-finishing and finishing, due to the higher discharge energy in roughing, which generates more molten material and therefore requires more effective flushing. Reducing the rotational speed to zero resulted in a high accumulation of carbon-based and oxygen-based phases on the machined surface due to reduced flushing performance. At very low rotational speeds, even suspended particles remaining in the dielectric from previous machining operations can adhere to the machined surface as a result of poor flushing.
- Increasing the rotational speed of the workpiece up to 45 rpm improved surface roughness by approximately 67% in roughing, 75% in semi-finishing, and 75% in the finishing regime. The enhanced flushing conditions at higher rotational speeds help remove debris and molten material from the machining gap, preventing them from solidifying inconsistently on the machined surface and thereby reducing surface coarseness.

## Figures and Tables

**Table 1:** The composition of WC-Co bars used as the workpiece.

Element	W	C	Co
W%	80.43	16.12	3.45

**Table 2:** The experimental details of the implemented EDT for the horizontal and vertical setups.

	Experience	Workpiece	Electrode	Rotational Speed (RPM)	Workpiece Diameter (mm)	Tool Electrode Diameter (mm)	Discharge Current (A)	Pulse On-Time (ms)	Pulse Off-Time (ms)	Voltage (V)
Horizontal Setup	1	47	11	5	6	12	5	300	20	80
	2	48	12	10	6	12	5	300	20	80
	3	49	13	20	6	12	5	300	20	80
					6	12				
Vertical Setup	4	46	23	5	6	12	5	300	20	80
	5	43	21	10	6	12	5	300	20	80
	6	45	22	20	6	12	5	300	20	80

**Table 3:** The experimental details of EDT with roughing, semi-finishing, and finishing regimes by the vertical setup.

	Test	Workpiece	Depth of Cut (mm)	Rotational Speed (rpm)	Workpiece Radius (mm)	Tool Electrode Radius (mm)	Discharge Current (A)	Pulse On-Time (µs)	Pulse Off-Time (µs)	Voltage (V)
Roughing	1	71	0.5	0 45	3	6	4	300	20	80
	2	72	0.5	0 45	3	6	4	300	20	80
	3	73	0.5	0 45	3	6	4	300	20	80
Semi-Finishing	4	71	0.3 0.2	0 45	3	6	4 2	300 150	20	80
	5	72	0.3 0.2	0 45	3	6	4 2	300 150	20	80
	6	73	0.3 0.2	0 45	3	6	4 2	300 150	20	80
Finishing	7	71	0.25 0.15 0.1	0 45	3	6	4 2 1	300 150 50	20	80
	8	72	0.25 0.15 0.1	0 45	3	6	4 2 1	300 150 50	20	80
	9	73	0.25 0.15 0.1	0 45	3	6	4 2 1	300 150 50	20	80

## Data Availability

No datasets were generated or analyzed during the current study.
